# Supplementary material for: An extended DNA-free intranuclear compartment organizes centrosome microtubules in malaria parasites
Source: Life Sci Alliance. 2021 Sep 17;4(11):e202101199. doi: 10.26508/lsa.202101199 (PMC8473725; doi:10.26508/lsa.202101199)
Supplement: Supplementary file 9 [file LSA-2021-01199_TableS1.docx]

**Table S1. List of primers used in this study**

| **For Plasmid** | **Name** | **Orientation** | **Sequence** |
| --- | --- | --- | --- |
| pArl-Centrin1-GFP | - | forward | CGACCCGGGATGGTACCATGAGCAGAAAAAATCAAACTATG |
| pArl-Centrin1-GFP | - | reverse | TTCTTCTCCTTTACTCCTAGGAAATAAGTTGGTCTTTTTCATAATTC |
| pSLI-Nup313-3xHA_glms | 0079 | forward | CGCAGCGCATCGCCTTCTATCGCCTTCTTGACGAGTTCTTCTAACTCGAGTAATTATAGCGCCCGAACTAAGCG |
| pSLI-Nup313-3xHA_glms | 0080 | reverse | GGTATAAATATATAAATAAGAAAAACGAACATTAAGCTGCCATATCCC |
| pSLI-Nup313-3xHA_glms | 0126 | forward | CCTTAGCTCATTCAGGTTTTTGTTTCGAAAAAATTATTACACCTGTGACGCGTTATCCATACGATGTTCCTGATTATGC |
| pSLI-Nup313-3xHA_glms | 0127 | reverse | GGATTTTCTTCTACATCTCCACATGTTAATAAACTTCCTCTTCCTTCTCCGTCGACAGCGTAATCAGGTACATCGTATGGATAAGAACC |
| pSLI-Nup313-3xHA_glms | 0163for | forward | ATAAGAATGCGGCCGCTAATGGATGTAATAAAAGTGATGATAGC |
| pSLI-Nup313-3xHA_glms | 0164rev | reverse | CGACGCGTATTTATCATATTTTGATTCATAAATTTATGCC |
| pSLI-Nup313-3xHA_glms | P22 | reverse | CGCTTCAGTGACAACGTCGAGCACAGC |
| pSLI-Nup313-3xHA_glms | P91 | forward | CACACAGGAAACAGCTATGACC |
| pSLI-Nup313-3xHA_glms | P248 | forward | AGATCTGATTCCATTTCTGG |
| pSLI-Nup313-3xHA_glms | P249 | reverse | GAGATAAGTAAGGATATACTTTTGC |
