# Supplementary material for: An extended DNA-free intranuclear compartment organizes centrosome microtubules in malaria parasites
Source: Life Sci Alliance. 2021 Sep 17;4(11):e202101199. doi: 10.26508/lsa.202101199 (PMC8473725; doi:10.26508/lsa.202101199)
Supplement: Supplementary file 10 [file LSA-2021-01199_TableS2.docx]

**Table S2. List of antibodies used in this study.**

| **Antibody** | **Species** | **Dilution*** | **Source** |
| --- | --- | --- | --- |
| anti-alpha-tubulin B-5-1-2, monoclonal (T5168) | mouse | 1:500 | Sigma |
| anti-alpha-tubulin TAT-1, monoclonal (00020911)** | mouse | 1:250 | Sigma |
| anti-beta-tubulin KMX-1, monoclonal (MAB3408)** | mouse | 1:250 | Sigma |
| anti-TgCentrin1, polyclonal | rabbit | 1:2000 | Marc-Jan Gubbels |
| anti-PfCentrin3, polyclonal | rabbit | 1:500 | This study |
| anti-CenH3, polyclonal | rabbit | 1:200 | Alan Cowman, (Volz et al., 2010) |
| anti-HA 3F10, monoclonal (12158167001) | rat | 1:500 | Sigma |
| anti-γ-Tubulin, polyclonal (T5192) | rabbit | 1:500 | Sigma |
| anti-mouse Alexa Fluor Plus 488** | goat | 1:100 | Thermo |
| anti-mouse-STAR580 | goat | 1:200 | Abberior |
| anti-mouse-Atto647 | goat | 1:200 | Sigma |
| anti-rabbit Alexa488 | goat | 1:1000 | Thermo |
| anti-rabbit Alexa Fluor Plus 488 | goat | 1:1000 | Thermo |
| anti-rabbit-Atto594 | goat | 1:200 | Sigma |
| anti-rabbit-Atto647 | goat | 1:200 | Sigma |
| anti-rat Alexa488 | goat | 1:500 | Thermo |
| anti-rat-Atto594 | goat | 1:200 | Biomol |
| RFP booster_Atto 594 | - | 1:200 | Chromotek |

* dilutions for regular IFAs; for U-ExM, antibodies were usually used two times more concentrated

** exclusively used for U-ExM; respective dilution/concentration corresponds to dilution for U-ExM
