# Supplementary material for: An extended DNA-free intranuclear compartment organizes centrosome microtubules in malaria parasites
Source: Life Sci Alliance. 2021 Sep 17;4(11):e202101199. doi: 10.26508/lsa.202101199 (PMC8473725; doi:10.26508/lsa.202101199)
Supplement: Supplementary file 11 [file LSA-2021-01199_TableS3.docx]

**Table S3. List of dyes used in this study**

| **Dye** | **Species** | **Concentration*** | **Source** |
| --- | --- | --- | --- |
| NHS ester-Atto 594 (AD 594-31)** | - | 10µg/ml | ATTO-TEC |
| SPY555-Tubulin (SC203) | - | 1:2000 | Spirochrome |
| 5-SiR-Hoechst | - | 1 µM for CLEM, 22 nM for live-cell | Jonas Bucevičius,  (Bucevičius et al., 2019) |
| Hoechst33342 | - | 1:1000 | Thermo |
| DRAQ5 | - | 1:1000 | Biostatus |

* concentrations for regular IFAs; for U-ExM, dyes were usually used two times more concentrated

** exclusively used for U-ExM; respective dilution/concentration corresponds to dilution for U-ExM
